# Supplementary material for: Effects of Molecular Structure on the Physical Properties of Fully Substituted Cellulose Esters of Aliphatic Acids
Source: Polymers (Basel). 2025 Apr 14;17(8):1053. doi: 10.3390/polym17081053 (PMC12030264; doi:10.3390/polym17081053)
Supplement: Supplementary file 1 [file polymers-17-01053-s001.zip › polymers-3438631-supplementary.pdf]

Supplementary Material

Effects of Molecular Structure on the Physical Properties of Fully Substituted Cellulose Esters of Aliphatic Acids

Taro Mori [1,2,*], Kanji Nagai [2,3] and Shu Shimamoto [2,4]

[1] Biomass Innovation Center, R&D Headquarters, Daicel Corporation, Kakuma-machi, Kanazawa-shi, Ishikawa 920-1192, Japan
[2] Graduate School of Natural Science and Technology, Kanazawa University, Kakuma-machi, Kanazawa-shi, Ishikawa 920-1192, Japan
[3] Life Sciences R&D Center, PharmaTek BU, Life Sciences SBU, Arai Plant, Daicel Corporation, Myoko-shi, Niigata 944-8550, Japan
[4] Business Development Center, R&D Headquarters, Daicel Corporation, Minato-ku, Tokyo 108-8230, Japan
* Correspondence: tr_mori@jp.daicel.com

Table S1. Feed amount of acid chlorides and DS of CEs.

CE		Acyl chloride[1]	Acetyl chloride		Substituent I	Substituent II	
ID	Name	equiv/AGU[2]	equiv/AGU[2]	Total amount	DS_x[3]	DS_{Ac}	DS_{total}
<u>SCE</u>							
CS3.0	Cellulose stearate	9.0	-	9.0	3.0	-	3.0
CP3.0	Cellulose palmitate	9.0	-	9.0	3.0	-	3.0
CM3.0	Cellulose myristate	9.0	-	9.0	3.0	-	3.0
CL3.0	Cellulose laurate	9.0	-	9.0	3.0	-	3.0
CH3.0	Cellulose hexanoate	9.0	-	9.0	3.0	-	3.0
CA3.0	Cellulose acetate	-	9.0	9.0	-	3.0	3.0
<u>MCE</u>							
CAL2.6	Cellulose acetate laurate	7.5	1.5	9.0	2.6	0.4	3.0
CAL2.1	Cellulose acetate laurate	6.0	3.0	9.0	2.1	0.9	3.0
CAL1.6	Cellulose acetate laurate	4.5	4.5	9.0	1.6	1.4	3.0
CAL1.0	Cellulose acetate laurate	3.0	6.0	9.0	1.0	2.0	3.0
CAL0.5	Cellulose acetate laurate	1.5	7.5	9.0	0.5	2.5	3.0

[1] Acyl chloride except for acetyl chloride.

[2] Stands for anhydroglucose unit.

[3] DS of acyl groups except for acetyl group.

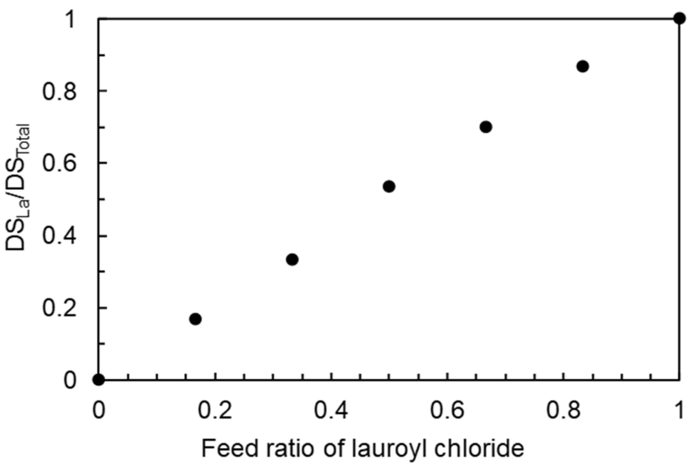

Figure S1. Relationship between the substituent ratio of the lauroyl group ($DS_{La}/(DS_{Ac}+DS_{La})$ and the feed ratio of the lauroyl chloride for the CA3.0, CL3.0, and CALs. The total amount of acyl chloride required was 9 mol for the three hydroxyl groups on each anhydroglucose unit.

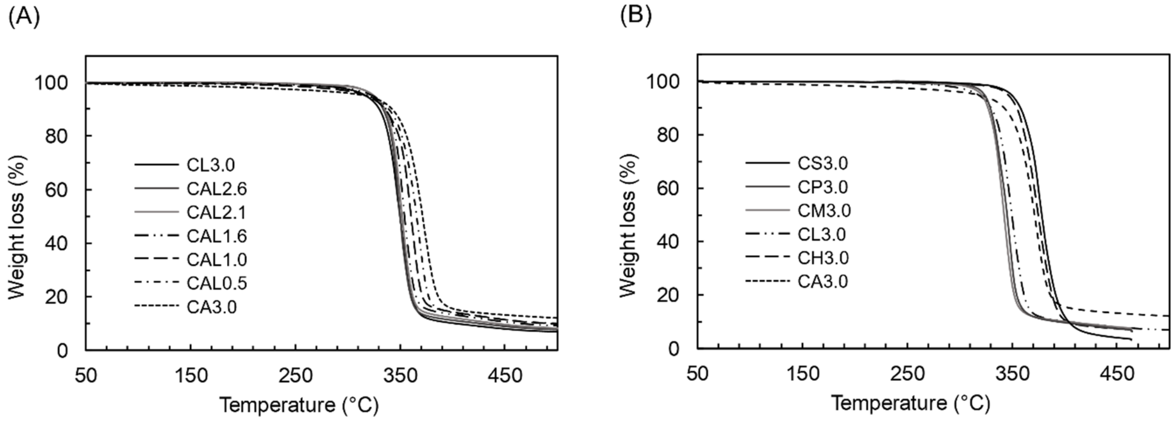

Figure S2. Thermogravimetric analysis (TGA) profiles of CEs upon heating from 50 °C to ~500 °C at a heating rate of 20 °C/min under a nitrogen atmosphere. (A) the CA3.0, CL3.0, and CALs; (B) SCEs (CA3.0, CH3.0, CL3.0, CM3.0, CP3.0, and CS3.0).

Table S2. DS, molar mass, and degree of polymerization of CEs prepared for the tensile test.

CE		Substituent I		Substituent II					Average molar mass[3]		
ID	Name	Carbon number	DS_x[1]	Carbon number	DS_{Ac}	DS_{Total}	DS_x/DS_{Total}	M_{AGU}[2] (g/mol)	$M_n/10^{-3}$ (g/mol)	$M_w/10^{-3}$ (g/mol)	DP_w[4]
CL3.0	Cellulose laurate	12	3.0	-	-	3.0	1.0	691	94	536	776
CAL2.3	Cellulose acetate laurate	12	2.3	2	0.4	2.7	0.9	612	85	254	415
CAL1.7	Cellulose acetate laurate	12	1.7	2	1.2	2.9	0.6	507	123	529	1043
CAL0.5	Cellulose acetate laurate	12	0.5	2	2.5	3.0	0.2	347	91	340	980

[1] DS of acyl groups except for acetyl group.

[2] Molar mass of anhydroglucose unit (repeat unit).

[3] Polystyrene equivalent number-average (M_n) or weight-average molecular weight (M_w) measured by GPC. Eluent: tetrahydrofuran (THF).

[4] Weight-average degree of polymerization defined as M_W/M_{AGU}.

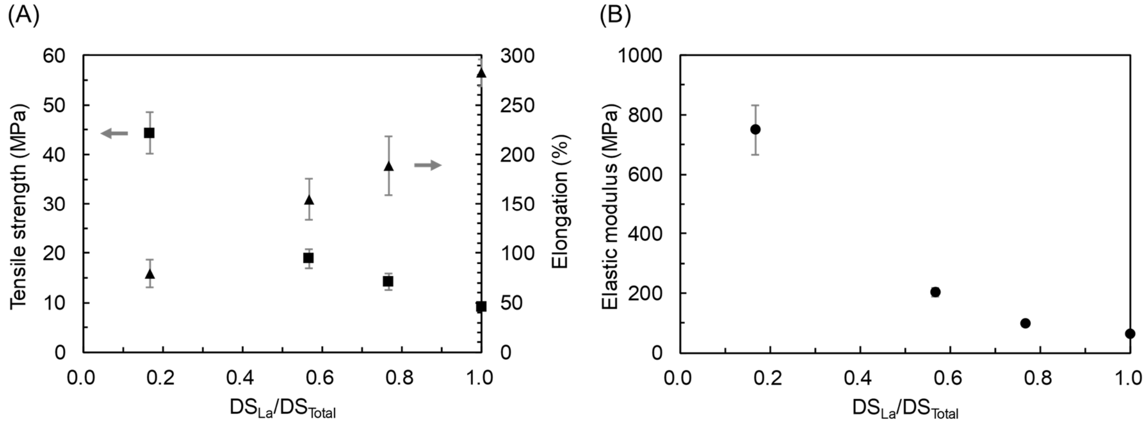

Figure S3. Mechanical properties of hot-pressed films prepared from the CL3.0 and CAL series. (A) Tensile strength: filled quadrangles. Elongation at break: filled triangles. (B) Elastic modulus: filled circles.

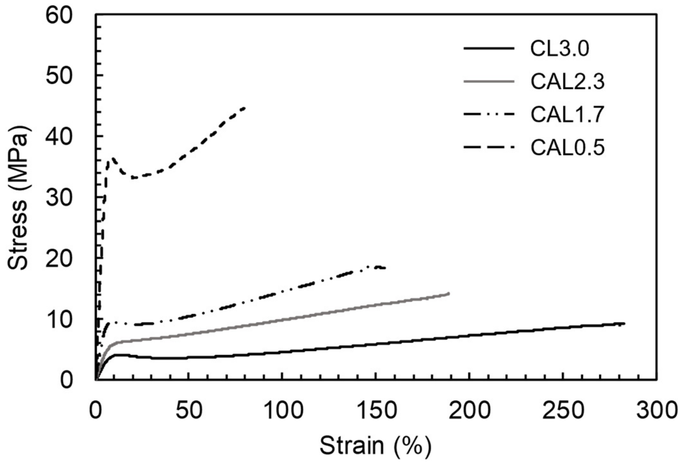

Figure S4. Stress-strain curves of hot-pressed films prepared from the CL3.0 and CALs.

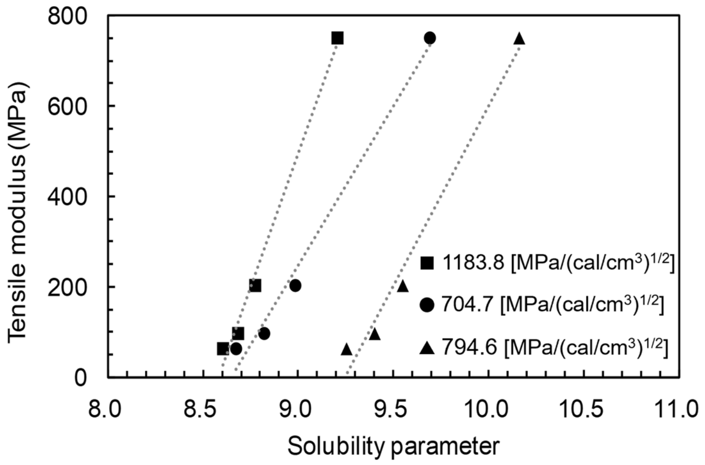

Figure S5. Tensile modulus values of the CA3.0, CL3.0 and CALs as a function of the calculated SP by means of group contribution methods. Method of Coleman [32]: filled circles. Method of Fedors [47]: filled triangles. Method of Hoftyzer-Van Krevelen [48]: filled quadrangles.
